# Metabolic Surgery on Patients With Polycystic Ovary Syndrome: A Systematic Review and Meta-Analysis

**DOI:** 10.3389/fendo.2022.848947

**Published:** 2022-03-10

**Authors:** Wenwen Yue, Xin Huang, Wenjing Zhang, Shumin Li, Xu Liu, Yian Zhao, Jiaxin Shu, Teng Liu, Weihua Li, Shaozhuang Liu

**Affiliations:** ^1^ School of Nursing and Rehabilitation, Cheeloo College of Medicine, Shandong University, Jinan, China; ^2^ Division of Bariatric and Metabolic Surgery, Department of General Surgery, Qilu Hospital of Shandong University, Jinan, China; ^3^ Center for Reproductive Medicine, Cheeloo College of Medicine, Shandong University, Jinan, China; ^4^ Key Laboratory of Reproductive Endocrinology of Ministry of Education, Shandong University, Jinan, China; ^5^ Cheeloo College of Medicine, Shandong University, Jinan, China; ^6^ Operating Theater, Qilu Hospital of Shandong University, Jinan, China

**Keywords:** polycystic ovary syndrome, metabolic surgery, bariatric surgery, obesity, meta-analysis, systematic review

## Abstract

Polycystic ovary syndrome (PCOS) is a complicated reproductive endocrine disease that is closely related to obesity. Metabolic surgery ameliorates a series of clinical manifestations and related comorbidities of PCOS. However, the overall efficacy of metabolic surgery on PCOS remains uncertain. This systematic review and meta-analysis aimed to evaluate the therapeutic effects of metabolic surgery on obese patients with PCOS. A systematic literature search for relevant studies was conducted on PubMed, Embase, Web of Science, and the Cochrane Library from inception to June 2021. Data extraction and quality evaluation were performed by three researchers, and RevMan 5.4 software was used to conduct the meta-analysis. A total of 14 studies involving 501 obese patients with PCOS were included. Incidence of PCOS in obese women ranged from 5.5% to 63.5% among the included studies. The results showed the incidence of abnormal menstruation decreased from 81% to 15% (OR=0.03, 95% confidence interval (CI): 0.01–0.08), while the incidence of hirsutism dropped from 71% to 38% (OR=0.21, 95% CI: 0.06–0.74). Serum total testosterone and free testosterone levels decreased by 25.92 ng/dL (MD = -25.92, 95% CI: -28.90– -22.93) and 2.28 ng/dL (SMD = -2.28, 95% CI: -3.67– -0.89), respectively. Sex hormone-binding globulin (SHBG) levels increased by 26.46 nmol/L (MD = 26.46, 95% CI: 12.97–39.95). Serum anti-Mullerian hormone (AMH) levels decreased by 1.29 ng/mL (MD = -1.29, 95% CI: -1.92– -0.66). Small sample size studies revealed that pregnancy rates ranged from 95.2% to 100% postoperatively. Metabolic surgery contributed to marked improvement of abnormal menstruation, hirsutism, and levels of free testosterone, total testosterone, SHBG, and AMH in patients with PCOS. Our findings indicate that patients with PCOS are expected to benefit from metabolic surgery, and could help potentially improve their reproductive outcomes. Metabolic surgery could thus be a new viable option for the clinical treatment of PCOS.

**Systematic Review Registration:**

PROSPERO https://www.crd.york.ac.uk/PROSPERO/, identifier CRD42021251524.

## 1 Introduction

Polycystic ovary syndrome (PCOS) is the most common endocrinopathy affecting women of reproductive age and is characterized by ovulation dysfunction, hyperandrogenism, and polycystic ovarian morphology (PCOM). The prevalence of PCOS ranges from 8% to 13% depending on the diagnostic criteria and study population (1, 2). Women with PCOS exhibit menstrual irregularities, hirsutism, and infertility. In addition, this syndrome can lead to long-term comorbidities, including obesity, type 2 diabetes mellitus, cardiometabolic diseases, endometrial carcinoma, and psychological disorders (3–5).

The pathogenesis of PCOS is multifactorial and remains unclear. However, a close relationship between obesity and the development of PCOS has been reported, with >50% of patients with PCOS as obese or overweight (6). In addition, hyperandrogenism, chronic inflammation, and family history of diabetes contributed to insulin resistance as the main pathogenetic mechanism of PCOS, with 50-70% of patients with PCOS exhibiting insulin resistance and compensatory hyperinsulinism (7, 8). Insulin resistance is considered an intrinsic feature of the syndrome, independent of body weight. However, obesity, a frequent feature among PCOS patients (60-70%), has a major contribution to the aggravation of insulin resistance (9). Studies have suggested that weight loss improves reproductive and metabolic dysfunction and reduces the risk of diabetes and cardiovascular disease (10, 11). Currently, lifestyle management targeting weight reduction is the first-line treatment for PCOS (12).

Metabolic surgery is the most effective and sustainable treatment for morbid obesity. Its therapeutic effects include resolution of type 2 diabetes, hypertension, hyperlipidemia, cardiovascular disease, and obstructive sleep apnea (13, 14). In addition, a few small-scale studies have indicated the positive effects of metabolic surgery on PCOS, including improvement of abnormal menstruation, hirsutism, fertility, and associated metabolic problems (10, 15). These findings suggested that metabolic surgery could be a potential treatment for PCOS; therefore, it has been recommended as an experimental therapy (2). However, there is no consensus on the use of metabolic surgery for the treatment of PCOS due to insufficient evidence. In the present study, we conducted a systematic review and meta-analysis to evaluate the therapeutic effects of metabolic surgery on PCOS.

## 2 Methods

This systematic review and meta-analysis was conducted and reported according to the Systematic Reviews and Meta-analyses guidelines (16, 17) (a checklist of the guidelines is provided in the **Supplementary Material**). A prespecified study protocol was developed and registered on PROSPERO (registration number: CRD42021251524).

### 2.1 Data Sources and Search Strategy

A comprehensive literature search was conducted on PubMed, Embase, Web of Science, and the Cochrane Library from their inception to June 2021. The search was focused on trials of metabolic surgery for treating PCOS and included the following search terms: (bariatric OR “metabolic surgery” OR “gastric bypass” OR “sleeve gastrectomy” OR “gastric banding”) AND (“polycystic ovary syndrome” OR “polycystic ovarian” OR “PCOS”). An email was forwarded to the corresponding author when there was no access to the full text. **Supplementary Table 1** lists the detailed search term.

### Eligibility Criteria

The inclusion criteria were as follows: (1) studies were randomized controlled trials (RCTs) or observational trials of metabolic surgery on patients with PCOS; (2) participants were diagnosed with obesity and PCOS and had undergone metabolic surgery; (3) participants were premenopausal women, with no restrictions for race or country; (4) studies should include at least ten patients, and study design had at least 6-month follow-up; (5) studies were published in English; and (6) studies reported at least one of the listed outcomes of interest–abnormal menstruation, hirsutism, and levels of total testosterone, free testosterone, anti-Mullerian hormone (AMH), and sex hormone-binding globulin (SHBG).

The exclusion criteria were as follows: (1) studies were in the form of abstracts, case reports, letters, comments, reviews, meta-analyses, and animal studies; (2) studies included no surgical intervention, lacked data on the outcome of interest; and (3) studies had unreliable designs or obvious statistical errors.

### 2.3 Study Selection and Data Extraction

Three independent reviewers (WY, WZ and XH) screened the titles and abstracts, conducted preliminary screening according to inclusion and exclusion criteria, further reviewed, screened, and cross-checked the full texts. Disagreement was settled by consensus.

The same reviewers extracted data independently into electronic data extraction forms, and discrepancies were resolved by consensus. Extracted data included information on study characteristics (author, publication date, country, study design, surgical procedure, duration of follow-up, sample size, PCOS cases), participant characteristics (age, body mass index [BMI]), and the outcomes mentioned above.

### 2.4 Quality Assessment and Risk of Bias

The methodological quality of RCTs was assessed using the Cochrane risk of bias tool (31). The quality of non-randomized studies was assessed using the MINORS scale (32). A higher score indicated a higher quality study. Disagreement among the reviewers was resolved by discussion. Funnel plots were examined to assess the publication bias.

### 2.5 Statistical Analysis

Statistical analyses were performed using Review Manager 5.4 (from the Cochrane Collaboration, http://www.cochrane.org). Odds ratios (OR) with 95% confidence intervals were calculated for dichotomous data. Mean difference (MD) or standardized mean difference (SMD) with 95% confidence intervals was calculated for continuous data. Heterogeneity was evaluated using Cochran’s Q test, and the degree of inconsistency (I^2^). I^2^ values <30%, 30% to 50%, and >50% were interpreted as low, moderate, and considerable heterogeneity, respectively. We performed the meta-analysis using a fixed-effect model if there was no significant heterogeneity among studies; otherwise, a random-effect model was applied, and sensitivity analysis was performed to confirm the validity of the analysis results. In this meta-analysis, the test level was set to α=0.05. Publication bias was evaluated using funnel plots.

## 3 Results

### 3.1 Search Results and Quality of Studies

**Figure 1:**
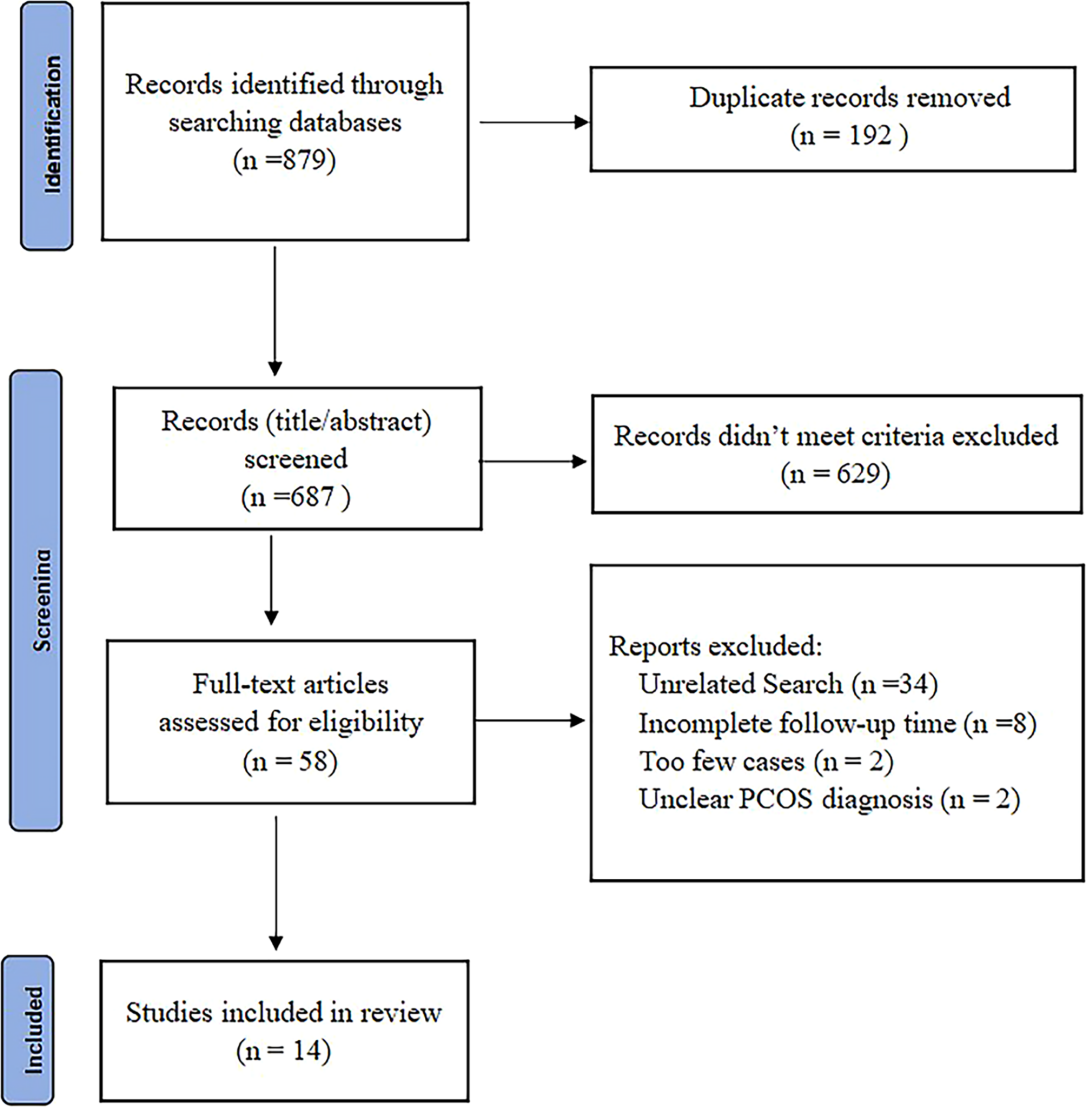
PRISMA diagram of the systematic review.

The study selection process is summarized **(** **Figure 1** **)**. From a total of 879 records, 192 were excluded due to duplication, 629 were excluded by title and abstract screening, and 44 were excluded after a further detailed review. A total of 14 studies conducted on 501 obese patients with PCOS were eligible and were in the meta-analysis (10, 18–30). After the quality assessment, no study was excluded because of lack of reliability **(** **Supplementary Table 2**).

### 3.2 Characteristics of Studies and Participants

The characteristics of the included studies are summarized **in** **Table 1**. These articles were published between 2005 and 2021. Overall, 3674 participants and 501 obese patients with PCOS were included in our analyses. Among the included studies, incidence of PCOS in obese women ranged from 5.5% to 63.5%.

**Table 1:** Baseline characteristics of included studies and participants.

Study	Country	Study design	Follow-up (mo)	Type of surgery	Total patients (n)	PCOS(n)	Age (yr)	Preop BMI(kg/m^2^)	Postop BMI(kg/m^2^)	EWL%	Outcomes extracted
Benito et al. (18)	Spain	Prospective	48	RYGB, SG, AGB	216	53	32.1 ± 5.3	44.6± 4.7	28.5 ± 4.1	–	②③⑥⑦
Bhandari et al. (19)	India	Prospective	6	SG	75	43	27.77 ± 4.50	42.52 ± 5.71	30.76± 2.93	58.37± 11.41	①⑤⑦
Casal et al. (10)	Spain	Retrospective	10.1 ± 1.5	LSG, LRYGB	217	43	33.19 ± 4.91	45.59 ± 4.97	27.73 ± 4.34		①
Chiofalo et al. (20)	Italy	Retrospective	12	SG, RYGB	55	29	30 ± 6	44 ± 7	–	65 ± 19	⑤⑦
Christ and Falcone, (21)	USA	Retrospective	22.8 ± 3.6	–	930	44	36.1 ± 2.3	44.2 ± 2.1	35.4 ± 1.5	–	②③⑦
Christinajoice et al. (22)	India	Retrospective	36	LSG, RYGB, LAGB	45	29	24.7 ± 10.2	41.5 ± 6.8		66.9 ± 24	④
Dilday et al. (23)	USA	Retrospective	12	SG	1385	119	31.5 ± 1.08	41.9 ± 5.2	29.55 ± 5.7	65.83 ± 21.8	⑦
Eid et al. (24)	USA	Retrospective	27.5 ± 16	LRYGB	24	24	34 ± 9.7	50 ± 7.5	30 ± 4.5	56.7	①④⑦
Eid et al. (25)	USA	Retrospective	12	LRYGB	14	14	36.3 ± 8.4	44.8 ± 1.6	29.2 ± 5.9	66.5	①②③④⑦
Escobar-Morreale et al. (26)	Spain	Prospective	12 ± 5	BPD, RYGB	36	17	29.8 ± 5.3	50.7 ± 7.1	41 ± 9	–	①②③⑦
Jamal et al. (27)	USA	Retrospective	46.7 ± 35.3	LRYGB	566	31	32 ± 5.8	52.8 ± 9.08	34.3 ± 5.7	64	④⑦
Singh et al. (28)	India	Prospective	12	LSG, LGB	50	18	29.7 ± 5.9	44.9 ± 7.5	–	63 ± 7.91	②④
Turkmen et al. (29)	Sweden	Prospective	6	RYGB	13	13	29.92 ± 7.12	47.15 ± 7.57	35.46 ± 7.04	–	①②⑥⑦
Wang et al. (30)	China	Prospective	12	LSG	48	24	25.5(22-35)	35.2(29-45.7)	31.7 ± 2.8	54 ± 24	①②⑦

BMI, body mass index; EWL%, excess weight loss percentage; SG, sleeve gastrectomy; RYGB, Roux-en-Y gastric bypass; AGB, adjustable gastric banding; LAGB, laparoscopic adjustable gastric banding; BPD, biliopancreatic diversion; LSG, laparoscopic sleeve gastrectomy; LRYGB, laparoscopic Roux-en-Y gastric bypass; LGB, laparoscopic gastric bypass; PCOS, polycystic ovary syndrome; ①, abnormal menstruation; ②, total testosterone; ③, free testosterone; ④, hirsutism; ⑤, AMH; ⑥, SHBG; ⑦, BMI.

### 3.3 Meta-Analysis Results

#### 3.3.1 Abnormal Menstruation

**Figure 2:**
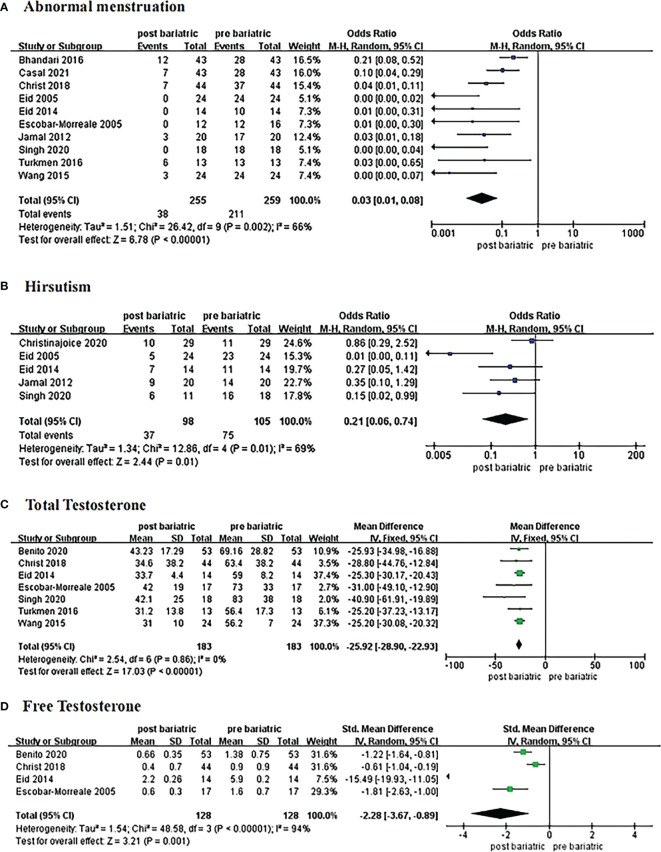
Forrest plots showing changes of abnormal menstruation **(A)**, hirsutism **(B)**, total testosterone **(C)**, and free testosterone **(D)** in women with PCOS after metabolic surgery.

Ten studies reported at least 6-month follow-up outcomes of abnormal menstruation (10, 19, 21, 24–30). Heterogeneity was considerable among studies (I^2^ = 66%, P=0.002); thus, the random-effects model was used for analysis. Meta-analysis results showed that metabolic surgery could reduce the incidence of menstrual abnormalities from 82% to 15% in women with PCOS [OR=0.03, 95%CI (0.01, 0.08), P<0.001] **(** **Figure 2A**).

#### 3.3.2 Hirsutism

Changes in hirsutism were reported in five studies with 105 patients (21, 24, 25, 27, 28). Due to the high heterogeneity (I^2^ = 69%, P<0.1), the random-effects model was employed. Data from the analysis revealed that metabolic surgery reduced the incidence of hirsutism from 71% to 38% [OR=0.21, 95%CI (0.06, 0.74), P=0.01] **(** **Figure 2B** **)**.

#### 3.3.3 Total Testosterone

Seven articles reported changes in total testosterone levels (18, 21, 25, 26, 28–30). The fixed-effects model was used for analysis because the heterogeneity was low (I^2^ = 0%, P>0.1). The results showed a decrease of 25.92 ng/dL in total testosterone in patients with PCOS after surgery [MD = -25.92, 95%CI (-28.90, -22.93), P< 0.00001] **(** **Figure 2C** **)**.

#### 3.3.4 Free Testosterone

Four studies contributed to the meta-analysis in terms of free testosterone levels (18, 21, 25, 26) in 128 women with PCOS. Heterogeneity was considerable (I^2^ = 94%, P<0.1) with regard to the measurement methods; hence, we adopted the standard mean difference (SMD) to summarize the data. The results of the meta-analysis showed that free testosterone in women with PCOS decreased by approximately 2.28 ng/dL after metabolic surgery [SMD = -2.28, 95%CI (-3.67, -0.89), P=0.001] **(** **Figure 2D** **)**.

#### 3.3.5 AMH

**Figure 3:**
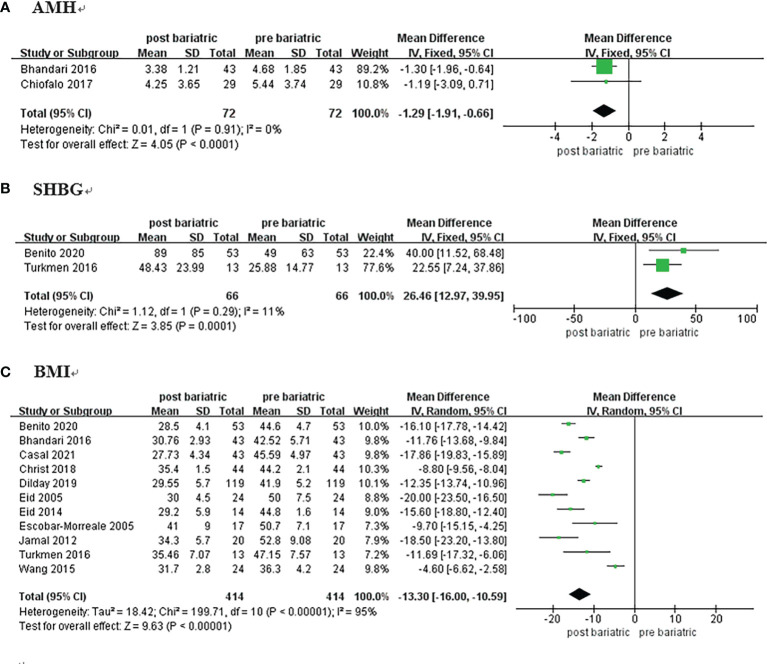
Forrest plots showing changes of AMH **(A)**, SHBG **(B)**, and BMI **(C)** in women with PCOS after metabolic surgery.

Two studies compared the pre and postoperative differences in AMH (19, 20); a total of 72 women with PCOS were included. As there was low heterogeneity (I^2 ^= 0%, P>0.1), the estimate was assessed with fixed-effects model. There was a significant difference between groups [MD = -1.29, 95%CI (-1.92, -0.66), P<0.00001)] **(** **Figure 3A** **)**, and the results showed that AMH decreased by approximately 1.29 ng/mL after surgery.

#### 3.3.6 SHBG

Two studies on 66 patients reporting pre and postoperative SHBG data were included (18, 29). The analysis was performed using a fixed-effects model because of low heterogeneity (I^2 ^= 11%, P=0.29). The results showed that SHBG in women with PCOS increased by approximately 26.46 nmol/L after metabolic surgery [MD = 26.46, 95%CI (12.97, 39.95), P = 0.001] (**Figure 3B**; **Table 2**).

**Table 2:** Summary for outcomes.

Outcomes	Number of studies	Number of cases	Heterogeneity test		Effect model	Meta-analysis results	
			I2(%)	P		Effect size (95%CI)	P
Abnormal menstruation	10	259	66	<.00001	REM	OR=0.03	<.00001
						[0.01, 0.08]	
Hirsutism	5	105	69	0.01	REM	OR=0.21	0.004
						[0.06, 0.74]	
Total testosterone	7	183	0	0.86	FEM	MD=-25.92	<.00001
						[-28.90,-22.93]	
Free testosterone	4	128	94	<.00001	REM	SMD=-2.28	0.001
						[-3.67, -0.89]	
AMH	2	72	0	0.91	FEM	MD=-1.29	<.00001
						[-1.91, -0.66]	
SHBG	2	59	0	0.46	FEM	MD=33.52	0.004
						[10.87, 56.17]	

CI, confidence interval; FEM, fixed effects model; MD, mean difference; OR, odds ratio; REM, random effects model; SMD, standardized mean difference.

#### 3.3.7 Body Mass Index

Eleven studies reported BMI among 414 patients (10, 18, 19, 21, 23–27, 29, 30). There was considerable heterogeneity detected (I^2^ = 95%, P<0.00001), and the meta-analysis was performed with the random-effects model. BMI of the patients decreased by approximately 13.30 kg/m^2^ after surgery [MD = -13.30, 95%CI (-16.00, -10.59), P< 0.00001] **(** **Figure 3C** **)**.

#### 3.3.8 Fertility and Pregnancy Outcomes

Three studies reported the reproductive outcomes of women after surgery and revealed that 31/32 patients with PCOS with a desire to conceive became pregnant after surgery (18, 24, 27). Jamal et al. (27) found that no pregnancy or postpartum complications were reported after surgery. The study by Benito et al. (18) showed that the live birth rates were 81.0% after surgery.

### 3.4 Publication Bias

**Figure 4:**
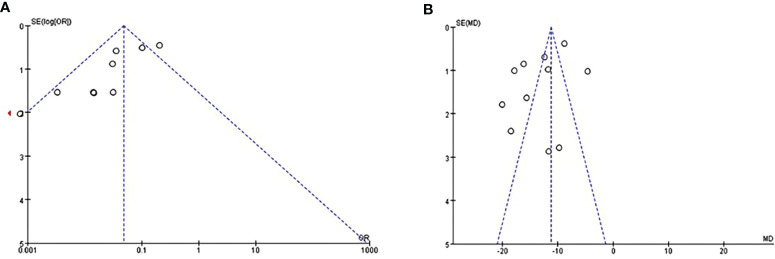
Evaluate of publication bias by funnel plots of studies reporting on abnormal menstruation **(A)** and BMI **(B)**.

Publication bias was assessed using funnel plots for two outcomes (abnormal menstruation and BMI) as the number of included studies was more than 10. The results suggested the presence of publication bias in these studies (**Figure 4**).

## 4 Discussion

This study is an updated systematic review and meta-analysis of the effects of metabolic surgery on PCOS. Fourteen high-quality studies including 501 patients were analyzed. The primary outcomes included changes in abnormal menstruation, hirsutism, and total and free testosterone levels. In addition, data on AMH and SHBG levels were integrated to gain a more comprehensive understanding of the beneficial effects of metabolic surgery. BMI and reproductive outcomes were also discussed. The principal findings of this study were as follows: (1) the incidence of abnormal menstruation decreased from 81% to 15% after metabolic surgery; (2) the incidence of hirsutism decreased from 71% to 38%; and (3) serum free testosterone, total testosterone, and AMH levels decreased, while SHBG levels increased postoperatively.

IR has been implicated in the pathogenesis of anovulation and infertility in PCOS, and abnormalities in insulin action have been observed in a variety of reproductive tissues in PCOS women (33). Obese women are more likely to have ovulatory dysfunction due to compensatory hyperinsulinism, hyperandrogenism and dysregulation of the hypothalamic-pituitary-ovarian axis, which manifests as sparse ovulation and menstrual abnormalities (34). Escobar-Morreale et al. (26) reported that weight reduction induced by Roux-en-Y gastric bypass (RYGB)/biliopancreatic diversion (BPD) contributed to the restoration of menstruation. In this study, the BMI decreased by 13.30 kg/m^2^, and the incidence of abnormal menstruation decreased from 81% to 15%. Two similar meta-analyses conducted previously revealed that the remission rates of abnormal menstruation were 49% and 77%, respectively (15, 35). Although metabolic surgery improved abnormal menstruation, a few patients still experienced menstrual irregularities after surgery. Therefore, predictors of improvement in abnormal menstruation after surgery need to be clarified.

Furthermore, hyperandrogenemia is considered a core pathophysiological feature of PCOS. It impairs follicular growth and maturation and causes abnormal menstruation, sparse ovulation, and hirsutism (36, 37). As previously suggested, obese patients are more prone to IR, and both obesity and IR represent the fundamental features of PCOS, which contribute to its pathogenesis and reinforce hyperandrogenemia (38). SHBG binds testosterone and reduces free testosterone levels in the blood (39). Women with PCOS and lower serum SHBG levels have a higher risk of developing hyperandrogenism, obesity, type 2 diabetes, metabolic syndrome, and cardiovascular disease (39, 40). Our study showed that SHBG levels increased by 26.46 nmol/L postoperatively, while free testosterone and total testosterone levels decreased by 2.28 ng/dL and 25.92 ng/dL, respectively. In addition, the incidence of hirsutism decreased from 71% to 38%. We considered that metabolic surgery was superior to medication for improving hyperandrogenemia. In a previous meta-analysis, SHBG levels increased by 7.8 nmol/L and free testosterone levels decreased by 1.77 ng/dL after metformin + GLP-1 receptor agonist treatment (41).

AMH is a glycoprotein produced by granulosa cells when follicle growth is initiated (42). Excessive production of AMH in the ovaries, which inhibits normal follicle growth, is considered an important feature of PCOS (43, 44). Elevated serum AMH levels lead to PCOM and oligomenorrhea among women with PCOS (45). The present study initially integrated data on AMH levels in patients after metabolic surgery, and the findings indicated a decrease of 1.29 ng/mL. Changes in PCOM were not analyzed due to the lack of data; only one study reported a decrease from 50% to 44% (21). Weight loss helps regulate serum AMH levels, improve PCOM, and restore menstruation (46). Therefore, further studies are required to determine the effects of metabolic surgery on AMH and PCOM in patients with PCOS.

The reproductive outcomes of patients in three studies were reviewed, and a significant improvement in pregnancy and fertility outcomes was noted postoperatively. In a review of six studies, Butterworth et al. (6) reported that pregnancy rates ranged from 33% to 100% after metabolic surgery in patients with PCOS. However, the sample size was very small (2–11), and the follow-up time was short. Hence, Further studies are needed to outline the advantages and disadvantages of metabolic surgery in terms of reproductive outcomes. According to a previous study, metabolic surgery reduced the risk of gestational diabetes, excessive fetal growth, and shorter gestation; however, it also increased the risk of small-for-gestational-age infants and stillbirth or neonatal death (47). Based on consensus recommendations, pregnancy should be postponed to 1 year after sleeve gastrectomy/RYGB, when a stable weight is achieved (48). For obese patients with PCOS who aim for fertility and are thus seeking metabolic surgery, the appropriate time to conceive needs further discussion.

Few studies have discussed the mechanisms that improve PCOS symptoms after metabolic surgery (49–51). In obese women, IR and hyperinsulinemia promote androgen secretion leading to hyperandrogenemia. Furthermore, serum SHBG and growth hormone levels decrease, while leptin and luteinizing hormone levels increase. Thus, neuroregulation of the hypothalamic-pituitary-ovarian axis is severely disturbed, and these factors affect the occurrence and progression of PCOS at multiple levels (52, 53). Although PCOS is known to improve with weight loss and that weight loss is effective in restoring IR, hyperandrogenism, and the hypothalamic-pituitary-ovarian axis (54–56), there is a possible weight-loss-independent mechanism for PCOS improvement after metabolic surgery. Eid et al. (25) reported that menstruation recovery occurred within a few weeks after surgery, while there was no significant weight loss, which is consistent with the clinical experience of our center. And improvements in PCOS were not correlated with the degree of weight loss. There is a weight-loss-independent mechanism of diabetes mellitus control after metabolic surgery that involves changes in gut hormones, bile acids, and gut microbiota (57). These factors are also associated with the pathogenesis of PCOS (49). Therefore, we believe that changes in gut hormones, bile acids, and gut microbiota contribute to the improvement of PCOS after metabolic surgery. Nonetheless, further studies are needed to elucidate the underlying mechanism.

There has been existing reviews and meta-analyses with the same topic (15, 35, 56). However, it did not impair the innovation or value of the present study. The major strength of this study is as follows: firstly, five extra newly published articles were included (10, 18, 20, 22, 23), the latest ones of which were published in 2020 (18, 22) and 2021 (10). Secondly, AMH and SHBG, two indicators closely related to PCOS, but never evaluated in any of the existing reviews and meta-analyses mentioned above, were assessed and discussed in the present study for the first time. Thirdly, we reviewed the pregnancy rates and fertility outcomes of patients with PCOS after metabolic surgery through three articles included in this meta-analysis (18, 24, 27). We also discussed the advantages and disadvantages of metabolic surgery on reproductive outcomes in patients with PCOS, as well as the optimal timing to conceive after surgery. Furthermore, all the articles included in the present study are with a follow-up time of >6 months and the number of PCOS cases>10, thus providing more credible and convincing evidence.

It should be noted that the present study had a few limitations. The heterogeneity was considerable in a part of outcomes which may resulted due to the differences in population characteristics, duration of follow-up, and diagnostic criteria for PCOS used among studies included. Moreover, potential publication bias was observed in the current study due to unpublished articles with negative results.

## 5 Conclusion

This study demonstrated that metabolic surgery significantly improved abnormal menstruation, hirsutism, and hyperandrogenism in women with PCOS. Serum AMH levels increased, and SHBG levels decreased postoperatively. Metabolic surgery may be a new viable treatment option for obese patients with PCOS. Further studies are required to confirm these beneficial effects and elucidate the underlying mechanisms.

## Data Availability Statement

The original contributions presented in the study are included in the article/**Supplementary Material**. Further inquiries can be directed to the corresponding authors.

## Author Contributions

WY, XH and WZ were major contributors in writing the Manuscript. WY, XH, and WZ contributed to literature search, screening, and data extraction. SLi, XL and YZ contributed to statistical analyses. JS and TL contributed to data validation. WL and SLiu. are responsible for review and modification of the manuscript. All authors read and approved the final manuscript.

## Funding

This study was supported by the Natural Science Foundation of Shandong Province (ZR201807290024), the Bethune Charitable Foundation (HZB-20190528-9), and the Clinical Research Center of Shandong University (2020SDUCRCC024).

## Conflict of Interest

The authors declare that the research was conducted in the absence of any commercial or financial relationships that could be construed as a potential conflict of interest.

## Publisher’s Note

All claims expressed in this article are solely those of the authors and do not necessarily represent those of their affiliated organizations, or those of the publisher, the editors and the reviewers. Any product that may be evaluated in this article, or claim that may be made by its manufacturer, is not guaranteed or endorsed by the publisher.
