# Supplementary material for: Metabolic Surgery on Patients With Polycystic Ovary Syndrome: A Systematic Review and Meta-Analysis
Source: Front Endocrinol (Lausanne). 2022 Mar 10;13:848947. doi: 10.3389/fendo.2022.848947 (PMC8961297; doi:10.3389/fendo.2022.848947)
Supplement: Supplementary file 1 [file DataSheet_1.pdf]

Supplementary Material

- Supplementary Table 1: Search strategy and detailed search term
- Supplementary Table 2: Quality assessment of studies included
- PRISMA Checklist

Supplementary Table 1 Search strategy and detailed search terms

Database	Search terms
PUBMED	(((((bariatric) OR ("metabolic surgery")) OR ("gastric bypass")) OR ("sleeve gastrectomy")) OR ("gastric banding"))<br>*AND*<br>((("polycystic ovary syndrome") OR (pcos)) OR ("polycystic ovarian"))<br>*Study design*<br>"controlled clinical trial" OR "randomized controlled trial" OR "groups" OR "trial" OR "randomly" OR "randomized"<br>*Limits*<br>NOT ("Animals" [Mesh])
EMBASE	bariatric OR 'metabolic surgery' OR 'gastric bypass' OR 'sleeve gastrectomy' OR 'gastric banding'<br>*AND*<br>'polycystic ovary syndrome' OR pcos OR 'polycystic ovarian'<br>*Study design*<br>'controlled clinical trial'/it OR 'randomized controlled trial'/it OR 'clinical trial'/it OR 'multicenter trial'/it OR 'controlled clinical trial'/exp OR 'randomized controlledtrial'/exp<br>*Limits*<br>Not ([animals]/lim AND [English]/lim
COCHRANE	bariatric:ti,ab,kw" OR "metabolic surgery:ti,ab,kw" OR "gastric bypass:ti,ab,kw" OR "sleeve gastrectomy:ti,ab,kw" OR "gastric banding:ti,ab,kw"<br>*AND*<br>"polycystic ovary syndrome:ti,ab,kw" OR pcos OR "polycystic ovarian:ti,ab,kw"
WEB OF SCIENCE	bariatric (Topic) or "metabolic surgery" (Topic) or "gastric bypass" (Topic) or "sleeve gastrectomy" (Topic) or "gastric banding" (Topic)<br>and<br>"polycystic ovary syndrome" (Topic) or pcos (Topic) or "polycystic ovarian" (Topic)

Supplementary Table 2. Results of the quality assessment of each included study using the "Methodological index for non-randomized studies" (MINORS) tool

study	A clearly stated aim	Inclusion of consecutive patients	Prospective collection of data	Endpoint appropriate to the aim of the study	Unbiased assessment of the study endpoint	Follow-up period appropriate to the aim of the study	Loss to follow up less than 5%	Prospective calculation of the study size	An adequate control group*	Contemporary groups*	Baseline equivalence of groups*	Adequate statistical analyses*	Total score
Benito et al.	2	2	2	2	2	2	0	0	2	2	2	2	20
Bhandari et al.	2	2	2	2	2	0	1	0	2	2	1	2	18
Casal et al.	2	2	2	2	2	2	0	0	2	1	1	2	18
Chiofalo et al.	2	2	1	2	2	2	2	0	2	2	1	2	20
Christ et al.	2	2	2	1	1	2	1	0	No control				11
Christinajoice et al.	2	2	2	2	1	2	0	0	No control				11
Dilday et al.	2	2	2	2	2	2	1	0	2	2	2	2	21

Supplementary Table 2. (continued) Results of the quality assessment of each included study using the "Methodological index for non-randomized studies" (MINORS) tool

study	A clearly stated aim	Inclusion of consecutive patients	Prospective collection of data	Endpoint appropriate to the aim of the study	Unbiased assessment of the study endpoint	Follow-up period appropriate to the aim of the study	Loss to follow up less than 5%	Prospective calculation of the study size	An adequate control group*	Contemporary groups*	Baseline equivalence of groups*	Adequate statistical analyses*	Total score
Eid et al.	2	1	2	1	0	2	0	2	No control				10
Eid et al.	2	2	1	1	2	2	0	0	No control				10
Escobar-Morreale et al.	2	2	2	2	2	2	0	0	1	2	1	2	18
Jamal et al.	2	2	1	1	2	2	0	0	No control				10
Singh et al.	2	2	2	1	2	2	0	0	No control				10
Turkmen et al.	2	2	1	1	1	1	2	0	No control				10
Wang et al.	2	2	2	2	2	1	0	0	2	2	1	2	18

* Additional criteria in the case of comparative studies

PRISMA checklist

Section/topic	#	Checklist item	Reported on page #
**TITLE**			
Title	1	Identify the report as a systematic review, meta-analysis, or both.	1
**ABSTRACT**			
Structured summary	2	Provide a structured summary including, as applicable: background; objectives; data sources; study eligibility criteria, participants, and interventions; study appraisal and synthesis methods; results; limitations; conclusions and implications of key findings; systematic review registration number.	1
**INTRODUCTION**			
Rationale	3	Describe the rationale for the review in the context of what is already known.	2
Objectives	4	Provide an explicit statement of questions being addressed with reference to participants, interventions, comparisons, outcomes, and study design (PICOS).	2-3<br>P – Patients with PCOS and obesity<br>I – metabolic surgery<br>C – none<br>O – characteristics related to PCOS<br>S – Comparative clinical trials (RCTs and OBSs)
**METHODS**			
Protocol and registration	5	Indicate if a review protocol exists, if and where it can be accessed (e.g., Web address), and, if available, provide registration information including registration number.	2
Eligibility criteria	6	Specify study characteristics (e.g., PICOS, length of follow-up) and report characteristics (e.g., years considered, language, publication status) used as criteria for eligibility, giving rationale.	2-3<br>Study Selection section

Information sources	7	Describe all information sources (e.g., databases with dates of coverage, contact with study authors to identify additional studies) in the search and date last searched.	2<br>Search Strategy section
Search	8	Present full electronic search strategy for at least one database, including any limits used, such that it could be repeated.	Table 1 in the Supplement
Study selection	9	State the process for selecting studies (i.e., screening, eligibility, included in systematic review, and, if applicable, included in the meta-analysis).	3<br>Study Selection section
Data collection process	10	Describe method of data extraction from reports (e.g., piloted forms, independently, in duplicate) and any processes for obtaining and confirming data from investigators.	3<br>Data Extraction section
Data items	11	List and define all variables for which data were sought (e.g., PICOS, funding sources) and any assumptions and simplifications made.	3
Risk of bias in individual studies	12	Describe methods used for assessing risk of bias of individual studies (including specification of whether this was done at the study or outcome level), and how this information is to be used in any data synthesis.	3<br>Quality Assessment section
Summary measures	13	State the principal summary measures (e.g., risk ratio, difference in means).	3
Synthesis of results	14	Describe the methods of handling data and combining results of studies, if done, including measures of consistency (e.g., I^2) for each meta-analysis.	3

**Section/topic**	**#**	**Checklist item**	**Reported on page #**
Risk of bias across studies	15	Specify any assessment of risk of bias that may affect the cumulative evidence (e.g., publication bias, selective reporting within studies).	3
Additional analyses	16	Describe methods of additional analyses (e.g., sensitivity or subgroup analyses, meta-regression), if done, indicating which were pre-specified.	3

**RESULTS**			
Study selection	17	Give numbers of studies screened, assessed for eligibility, and included in the review, with reasons for exclusions at each stage, ideally with a flow diagram.	3-4<br>Search Results section<br>Flow chart was shown in Figure 1.
Study characteristics	18	For each study, present characteristics for which data were extracted (e.g., study size, PICOS, follow-up period) and provide the citations.	4<br>Results detailed in Table 1
Risk of bias within studies	19	Present data on risk of bias of each study and, if available, any outcome level assessment (see item 12).	5<br>Results detailed in Table 2
Results of individual studies	20	For all outcomes considered (benefits or harms), present, for each study: (a) simple summary data for each intervention group (b) effect estimates and confidence intervals, ideally with a forest plot.	4-5<br>Described in Results sections Meta-analysis on changes in patients with PCOS pre-and postoperative
Synthesis of results	21	Present results of each meta-analysis done, including confidence intervals and measures of consistency.	4-5<br>Described in Results sections Meta-analysis on changes in patients with PCOS pre-and postoperative
Risk of bias across studies	22	Present results of any assessment of risk of bias across studies (see Item 15).	5<br>Results detailed in Figure 3 in the Supplement
Additional analysis	23	Give results of additional analyses, if done (e.g., sensitivity or subgroup analyses, meta-regression [see Item 16]).	4
**DISCUSSION**			
Summary of evidence	24	Summarize the main findings including the strength of evidence for each main outcome; consider their relevance to key groups (e.g., healthcare providers, users, and policy makers).	5-6

Limitations	25	Discuss limitations at study and outcome level (e.g., risk of bias), and at review-level (e.g., incomplete retrieval of identified research, reporting bias).	6-7
Conclusions	26	Provide a general interpretation of the results in the context of other evidence, and implications for future research.	7
**FUNDING**			
Funding	27	Describe sources of funding for the systematic review and other support (e.g., supply of data); role of funders for the systematic review.	Not applied

From: Moher D, Liberati A, Tetzlaff J, Altman DG, The PRISMA Group (2009). Preferred Reporting Items for Systematic Reviews and Meta-Analyses: The PRISMA Statement. PLoS Med 6(7): e1000097. doi:10.1371/journal.pmed100009
